# COVID-19 Pandemic Worry and Vaccination Intention: The Mediating Role of the Health Belief Model Components

**DOI:** 10.3389/fpsyg.2021.674018

**Published:** 2021-07-12

**Authors:** Claudia I. Iacob, Daniela Ionescu, Eugen Avram, Daniel Cojocaru

**Affiliations:** ^1^Laboratory of Health Psychology and Clinical Neuropsychology, Department of Psychology, Faculty of Psychology and Educational Sciences, University of Bucharest, Bucharest, Romania; ^2^Department of Psychology, Faculty of Political Sciences, National University of Political Studies and Public Administration, Bucharest, Romania

**Keywords:** COVID-19, pandemic worry, vaccination intention, health belief model, chronic illness

## Abstract

Given the negative consequences of the ongoing COVID-19 pandemic on public health, his study aimed at investigating: (1) the differences between adults with and without chronic illness in buying behavior, vaccination intention, pandemic worry, and the health belief model (HBM) components; (2) the HBM components as mediators of the relationship between pandemic worry and vaccination intention. The sample consisted of 864 adults (66.6% females, *M*_age_ = 47.61, *SD* = 9.23), of which 20.5% reported having a chronic illness. Associations between pandemic worry, vaccination intention, and HBM were ascertained using correlation and mediation analyses. Individuals with chronic illness reported a higher level of pandemic worry, higher levels of perceived threat, greater benefits from vaccination, had lower self-efficacy and bought more medicine and sanitary/hygienic products. No significant differences were observed regarding vaccination intention, barriers against vaccination, and changes in food buying behavior. We found that the relationship between pandemic worry and vaccination intention was partially mediated by the perceived threat of disease and the benefits of vaccination. Pandemic worry predicted vaccination intention directly but also through the contribution of the perceived threat of disease and the benefits of vaccination. These findings suggest that presenting evidence of COVID-19 vaccine efficacy and the benefits of having the vaccine (especially for vulnerable groups, such as chronic illness patients) will encourage the population to follow vaccination recommendations.

## Introduction

Vaccination is one of the central preoccupations during the current COVID-19 pandemic, as it strikes the world rapidly and pandemic worry spreads around the globe (World Health Organization, 2020). The increasing infection and mortality rates, especially in vulnerable populations, such as chronic illness patients, revealed a preoccupation for treatment optimization, especially for a vaccine with high uptake in the population (Centers for Disease Control and Prevention, 2020b). The high interest in vaccination is argued by the impact of the ongoing COVID-19 pandemic in various areas relevant for the current discussion: economy, psychological functioning, and psychosocial consequences (Bashir et al., 2020; Norouzi et al., 2020).

Vaccination intention was intensively studied considering the continuous rising of the Anti-vaxxers movement in the last years and the increasing number of people refusing vaccination lately (Greenberg et al., 2019). The literature investigated factors that can influence the decision to vaccinate for various diseases. The following factors were positively associated with vaccination intention: pandemic worry and perceived threat of disease (Ashbaugh et al., 2013; Liao et al., 2013), a habit for seasonal influenza vaccination (Schmid et al., 2017), confidence in the safety of the vaccine and the information provided by the authorities, social comparisons with people who want to receive the vaccine (Podlesek et al., 2011), old age, a very high level of education, also a very low level of education (Bonfiglioli et al., 2013), being part of social categories exposed to risk infection (Bish et al., 2011).

Most studies regarding vaccination intention are based upon two prevalent theoretical frameworks: Theory of Planned Behavior (Gallagher and Povey, 2006) and health belief model (HBM; Cummings et al., 1979). These models explained almost 60% of the variance in young women’s vaccination intentions against Human Papillomavirus (HPV) (Bennett et al., 2012). The HBM is one of the models used most extensively in health behavior research (Skinner et al., 2015). The original model has four components: (1) perceived susceptibility of disease (i.e., the perceived probability of contracting the disease/infection); (2) perceived severity (i.e., how bad are the consequences of the disease); (3) perceived benefits of preventive actions or treatment; (4) perceived barriers in carrying out the recommendations regarding preventive actions (Janz and Becker, 1984). Later on, other components were added (e.g., demographics, perceived control, self-efficacy) (DiClemente and Peterson, 1994). It has been successfully used as a framework to predict vaccination intentions for seasonal influenza in children (He et al., 2015) and young adults (Fall et al., 2018).

The HBM component connected to intention throughout multiple studies is the perceived threat of disease, which refers to the perceived risk of potential illness and its consequences on individual health (Liao et al., 2013). Pandemic worry is an emotional response regarding the disease (Ro et al., 2017), and it includes the perception of potential risk for infection, the risk for the family to become infected, the perceived severity of the disease, and the consequences on one’s health (Goulia et al., 2010). It is closely related to risk perception and people’s preventive behavior in a pandemic crisis (Goulia et al., 2010), and that is why it was considered relevant for this paper’s scope.

Just as in previous major health crises, the population engaged in safety and preventive measures recommended or reinforced by their governments (Liu et al., 2020) and different shopping patterns that unbalanced store supplies (Sim et al., 2020). Given all these efforts to adapt, behavioral science contributes by exploring psychosocial responses connected with health behaviors.

Chronic diseases or illnesses are long-term diseases that affect the life and daily functioning of the person for at least one year and require continuous or periodic medical management (Centers for Disease Control and Prevention, 2020a). There are several types of chronic diseases, depending on the affected system (e.g., cardiovascular, respiratory, neurological, digestive). This study considered cardiovascular disease, respiratory disease, diabetes, and two psychiatric disorders (anxiety and depression). People with chronic illnesses (i.e., cardiovascular, respiratory, diabetes, and cancer) are more prone to develop severe COVID-19 related symptoms and have an increased mortality rate than the general healthy population (Jordan et al., 2020). As such, for maintaining social balance, it is essential to explore people’s reactions in the first stages of the COVID-19 pandemic, thus helping to establish a correct pattern of action in future situations like this.

The present study subscribes to the HBM to explain the vaccination intention. This model is already used to explain the relationship between pandemic worry and vaccination intention during the H1N1 pandemic. The perceived threat of disease, benefits, and barriers of vaccinations mediated the association (Scherr et al., 2016).

As such, this paper has two objectives: (1) to explore the differences between adults with and without chronic illness in buying behavior, vaccination intention, pandemic worry, and the HBM components; (2) to examine the HBM components as mediators of the relationship between pandemic worry and vaccination intention. For the first objective, we expect people with chronic illness to buy more supplies, to have greater pandemic worry levels, and to have a greater intention to vaccinate when compared with people without chronic illness. Regarding the HBM components, we expect them to have higher levels of threat perception and benefits from vaccination, but lower levels of barriers and self-efficacy, due to the perceived sense of vulnerability a chronic illness installs.

## Materials and Methods

### Participants and Design

This cross-sectional design study is based on a convenience sample of 864 Romanian community adults (66.6% females), with ages ranging between 31 and 65 (*M* = 47.61, *SD* = 9.23). 20.5% reported having a chronic illness (e.g., cardiovascular disease, respiratory disorder, diabetes). The study included Romanian adults living in Romania during the COVID-19 state of emergency, able to give informed consent. We used the following exclusion criteria: adults under 30 years old, previous or current diagnosis of COVID-19.

### Measures

Vaccination intention was measured with one item: “Do you intend to get vaccinated when offered a vaccine against COVID-19 infection?” The answers were coded from 1 to 3, as follows: 1 (*no*), 2 (*maybe*), and 3 (*yes*).

*Pandemic Worry*. The worry frequency and severity regarding the COVID-19 pandemic were measured using an adapted version of the Dispositional Pandemic Worry Scale (Scherr et al., 2016), initially conceived for the H1N1 flu pandemic of 2009–2010. Answers were rated on a 6-point Likert scale, from 1 (*not at all*) to 6 (*very much*). All items were scored directly. Items 1–4 addressed the worrying frequency, while items 5–8 addressed the worry severity. For this scale, the Cronbach α index of internal consistency was very good, α = 0.92.

*The HBM Components*. The perceived threat of disease, benefits, barriers, and self-efficacy regarding vaccination are the four main components of the HBM model investigated in this research. The perceived threat of disease was assessed with a 4-item scale adapted after Champion (1999) instruments which measured HBM components. The Cronbach alpha index was α = 0.77. The benefits of vaccination were evaluated with a 5-item scale adapted from Champion. For this scale, the Cronbach alpha index was α = 0.87. Barriers to vaccination were examined with a 10-item scale adapted from the same source. The Cronbach Alpha index for the barriers scale was α = 0.81. Self-efficacy regarding COVID-19 infection was analyzed with a 5-item scale adapted from Champion et al. (2005) instrument regarding self-efficacy for mammography. The Cronbach alpha index for this scale is α = 0.76. All the answers to HBM components were scored on a 5-point Likert scale, from 1 (*highly unlikely*) to 5 (*most likely*), and all the items were scored directly.

*Changes in buying behavior* were investigated using three questions regarding the amount of food, medication, and hygienic-sanitary items the participants purchased since the declaration of the COVID-19 pandemic. The answers were scored on a 10-point scale, ranging from 1 (*I buy as usual*) to 10 (*I buy ten times more than the usual amount*).

Socio-demographic data were obtained through a questionnaire inquiring about gender, age, education level, and chronic illness.

### Procedure

The questionnaires were shared in online and social media environments during the state of emergency declared by the Romanian government. The Google Form questionnaire was available from March until May 2020. The participants read and agreed to an informed consent that provided information regarding the aims of the study, procedures, confidentiality (GDPR), and the possibility of withdrawing from the study at any moment, without consequences. Also, they could contact the researchers via e-mail for additional information. The study was conducted following the Declaration of Helsinki.

### Data Analysis

All the data analyses were performed using the statistical software JAMOVI, version 1.1.9. We reported the main descriptive statistics (mean, standard deviation, frequencies, Pearson chi-square values of group differences). We used skewness and kurtosis indicators with values between −1.96 and +1.96 to establish the normality of the data distribution (George and Mallery, 2010). For examining the differences between participants without chronic illness (group 1) and participants with chronic illness (group 2), we used Welch’s *t*-test on normally distributed variables and Mann-Whitney *U* test for non-normally distributed data. A positive mean difference reflected higher scores reported by group 1, and a negative mean difference was indicative of higher scores reported by group 2. Cohen’s *d* coefficient was used (Cohen, 1988) to depict the magnitude of the effect size in the mean difference.

We used principal component analysis to investigate the factor structure of the adapted measures to determine if the items cluster into one or more factors that explained as much as possible of the overall variance (Sava, 2011). We conducted the Bartlett’s test of sphericity and the Kaiser-Meyer-Olkin index of sampling adequacy to see whether the data is suitable for structure detection. A significant value for the first test and a value closer to 1 for the second test were considered acceptable in terms of usefulness of factor analysis. Also, we reported the cumulative variance explained by the items and the factor loadings.

Applying a general linear mediation model (i.e., GLM mediation model), we tested the mediation role of HBM components (i.e., perceived threat, benefits, barriers, and self-efficacy) on the relationship between pandemic worry and vaccination intention. We examined the direct, indirect, and total effects of pandemic worry and HBM components on vaccination intention. We used the jAMM module, which applies the maximum likelihood estimation method, an optimal procedure for parameter estimations. Using the Delta method, which extends the approximations from the central limit theorem (Deng et al., 2018), we calculated the confidence intervals.

## Results

### Socio-Demographic Information and Descriptive Statistics

The sample’s socio-demographic characteristics are in Supplementary Table 1. The main descriptive statistics and the correlations between the variables are depicted in Table 1. The skewness and kurtosis indicators had acceptable values, ranging between (−1.96 and 1.96) for all variables, except for medicine buying. Vaccination intention had significant correlations with all the studied variables, except for education level and self-efficacy. The strongest correlations were with benefits (*r* = 0.68, *p* < 0.001) and barriers to vaccination (*r* = −0.60, *p* < 0.001), in the expected direction. Pandemic worry correlated with all the variables, except for barriers to vaccination. The strongest association was with the perceived threat of disease (*r* = 0.49, *p* < 0.001). Supplementary Table 2 presents the frequencies for vaccination intention and changes in buying food, medicine, and sanitary/hygienic supplies.

**TABLE 1:** Descriptive statistics and correlations between study variables.

	**1**	**2**	**3**	**4**	**5**	**6**	**7**	**8**	**9**	**10**	**11**
1	–										
2	0.21***	–									
3	0.33***	0.49***	–								
4	0.68***	0.28***	0.37***	–							
5	−0.60***	0.02	−0.17***	−0.47***	–						
6	0.001	−0.22***	−0.25***	0.06	0.01	–					
7	0.08*	0.30***	0.22***	0.12***	0.03	−0.10**	–				
8	0.07*	0.35***	0.26***	0.09**	0.03	−0.16***	0.57***	–			
9	0.12***	0.35***	0.26***	0.14***	0.08*	−0.09**	0.55***	0.59***	–		
10	0.08*	0.11**	0.01	0.07*	0.01	0.009	–0.04	0.01	0.06	–	
11	0.03	−0.08*	0.04	0.04	−0.07*	0.02	0.05	–0.01	0.05	−0.09**	–
Mean	2.23	17.00	9.68	16.1	20.9	18.7	2.66	2.08	3.01	47.61	–
SD	0.75	8.69	3.47	5.79	7.51	4.12	1.85	1.77	2.20	9.23	–
Skewness	–0.40	1.25	0.35	–0.32	0.85	–0.44	1.34	2.13	1.27	0.44	−0.44
Kurtosis	–1.13	1.25	–0.43	–0.77	0.54	0.08	1.64	4.42	1.10	–0.59	−0.73

1, vaccination intention; 2, pandemic worry; 3, perceived threat; 4, benefits; 5, barriers; 6, self-efficacy; 7, food buying; 8, medicine buying; 9, hygienic/sanitary buying; 10, age, 11, education.

*p < 0.05, **p < 0.01, ***p < 0.001.

### Exploratory Factor Analyses for the Adapted Measures

All the adapted instruments had skewness and kurtosis indicators within acceptable range and the main assumptions for exploratory factor analysis were met. The results revealed that the items explained between 53.9 and 80.5% of the scales’ total variance, with high loadings of most items, thus providing evidence of the internal reliability of the measures. For details, consult Supplementary Table 3.

### Differences Among Adults With and Without Chronic Illness

Participants with chronic illness reported a higher level of pandemic worry [*t*(249) = −6.33, *p* < 0.001, *d* = −0.58], higher levels of perceived threat [*t*(259) = −0.95, *p* < 0.01, *d* = −0.27], greater benefits from vaccination [*t*(286) = −1.07, *p* = 0.02, *d* = −0.18] and lower self-efficacy [*t*(280) = 2.44, *p* = 0.01, *d* = 0.20]. Regarding changes in buying behavior, people with chronic illness bought more medicine (*U* = 52152, *p* < 0.001, *d* = 0.14) and sanitary/hygienic products [*t*(247) = −2.60, *p* = 0.01, *d* = −0.24]. No significant differences were observed regarding vaccination intention, barriers against vaccination, and changes in food buying behavior. All the results are presented in Table 2.

**TABLE 2:** Group differences between participants without chronic illness and with chronic illness.

	**Test value**	***df***	**Mean difference**	**SE**	***p*-value**	***d***
Vaccination intention	−1.59	287	−0.09	0.06	0.11	−0.12
Pandemic worry	−6.33	249	−4.91	0.77	<0.001	−0.58
Perceived threat	−3.13	259	−0.95	0.30	<0.01	−0.27
Benefits	−2.28	286	−1.07	0.47	0.02	−0.18
Barriers	0.001	294	0.001	0.60	0.99	0.00
Self-efficacy	2.44	280	0.83	0.34	0.01	0.20
Food supplies	−1.67	253	−0.28	0.16	0.09	−0.15
Medicine supplies	52152	–	−5.70e-5	0	<0.001	0.14
Sanitary supplies	−2.60	247	−0.52	0.20	0.01	−0.24

### The Health Belief Model Components as Mediators of the Relationship Between Pandemic Worry and Vaccination Intention

**FIGURE 1:**
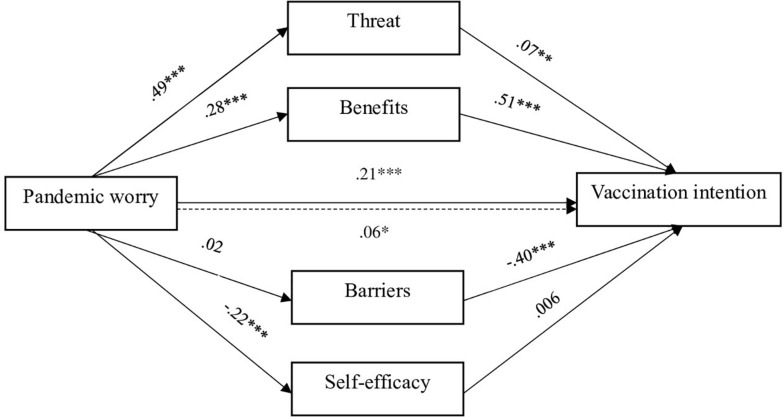
Path diagram of the GLM mediation, with β coefficients. *p < 0.05; **p < 0.01; ***p < 0.001.

The total effect of pandemic worry on vaccination intention was significant [β = 0.21, *p* < 0.001, 95% CI (0.01,0.02)]. The direct effect of pandemic worry on vaccination intention was significant [β = 0.06, *p* = 04, 95% CI (0.001,0.009)] but smaller than the total effect, indicating partial mediation effects. As presented in Table 3, the perceived threat of disease and benefits of vaccination were mediators of the relationship, as the indirect effects and the components’ regression coefficients were significant. Barriers against vaccination and self-efficacy did not mediate the relationship between pandemic worry and vaccination intention. The path diagram of the GLM mediation model, with the β coefficients, is displayed in Figure 1. To check whether the non-significant results are due to a lack of statistical power, we performed *post hoc* power analysis using the software Quantpsy.org (Preacher and Coffman, 2006). For α = 0.05, at a sample size of *N* = 864 and df = 3, we obtained a statistical power of 0.94, indicating high power. As such, it is unlikely that the non-significant findings can be attributed to small sample size.

**TABLE 3:** Direct, indirect, and total effects of the GLM mediation.

**Type**	**Effect**			**95% C.I.^a^**				
		**Estimate**	**SE**	**Lower**	**Upper**	**β**	**z**	***p***
Indirect	Panworry ⇒ Threat ⇒ Intentvaccin	0.003	0.001	0.001	0.005	0.03	2.76	0.006
	Panworry ⇒ Benefits ⇒ Intentvaccin	0.01	0.001	0.008	0.01	0.14	8.03	<0.001
	Panworry ⇒ Barier ⇒ Intentvaccin	−0.001	0.001	−0.002	0.001	−0.01	−0.8	0.42
	Panworry ⇒ Self-efficacy ⇒ Intentvaccin	−0.001	0.001	−0.001	0.001	−0.001	−0.26	0.79
Component	Panworry ⇒ Threat	0.19	0.01	0.17	0.21	0.49	16.56	<0.001
	Threat ⇒ Intentvaccin	0.01	0.005	0.004	0.02	0.07	2.8	0.005
	Panworry ⇒ Benefits	0.19	0.02	0.14	0.23	0.28	8.76	<0.001
	Benefits ⇒ Intentvaccin	0.06	0.003	0.05	0.06	0.51	20.09	<0.001
	Panworry ⇒ Barrier	0.02	0.02	−0.03	0.08	0.02	0.8	0.42
	Barrier ⇒ Intentvaccin	−0.03	0.002	−0.04	−0.03	−0.4	−16.57	<0.001
	Panworry ⇒ Self-efficacy	−0.1	0.01	−0.13	−0.07	−0.22	−6.64	<0.001
	Self-efficacy ⇒ Intentvaccin	0.001	0.004	−0.007	0.009	0.006	0.26	0.79
Direct	Panworry ⇒ Intentvaccin	0.004	0.002	0.001	0.009	0.06	2.02	0.04
Total	Panworry ⇒ Intentvaccin	0.01	0.002	0.01	0.02	0.21	6.33	<0.001

*^a^Confidence intervals computed with method: Standard (Delta method).*

## Discussion

Amid the ongoing COVID-19 pandemic, preventive measures, vaccination, and pandemic worry are topical due to their importance for public policymaking. That is the reason why, in the current study, we aimed to investigate two significant aspects related to these concepts: (1) the differences between adults with and without chronic illness in buying behavior, vaccination intention, pandemic worry, and the HBM components; (2) the HBM components as mediators of the relationship between pandemic worry and vaccination intention.

Regarding the first objective, data showed that participants with chronic illness displayed higher levels of pandemic worry, higher levels of the perceived threat, and reported greater benefits from vaccination than healthy participants. This result is expected considering that participants are exposed to informational sources (e.g., television, newspapers, brochures) that highlight the liability of chronic illness patients when faced with COVID-19. Thus, this installs a sense of vulnerability in front of a potential infection. According to this data, even if the first group reported greater benefits from vaccination, this perception was not associated with a greater intention to vaccinate. This finding has negative implications for the success of a future vaccination campaign, as intention predicts behavior (Sheeran, 2002). Additionally, vaccination uptake is suboptimal in many countries, including Romania (Habersaat et al., 2020).

Regarding changes in shopping behavior, people with chronic illness bought more medicine and sanitary/hygienic products than people without chronic illness. Their normal functioning depends on the continuation of treatment for heart disease or diabetes. As such, they are expected to buy larger supplies of medicines during times of health crisis. This result is consistent with the previous one, reflecting the vulnerability of patients with chronic illness. There was no significant difference concerning food buying. Overall, participants bought 2.66 times more food during the state of emergency. A recent review highlighted that the psychological causes for panic buying are related to the people’s perceived threat of the crisis and scarcity of supplies, people’s fear of the unknown and uncertainty, coping styles, and the social influence of others (Yuen et al., 2020). According to neuroscience, gathering enormous amounts of food is how evolution has taught us to manage periods of resource shortage. Therefore, it is deeply rooted in our brains to have extra supplies in times of crisis. Buying more sanitary hygienic products during pandemics (e.g., hand sanitizer, toilet paper) is an attempt to avoid diseases and is motivated by safety concerns and disgust regarding germs (Taylor, 2019).

A primary objective of this study was to assess a particular set of latent psychological constructs that could lead to a better understanding of why those differences between healthy individuals and those diagnosed with a chronic illness can lead to distinct behavioral responses. In this research, there was no significant difference in vaccination intention between the two groups. In contrast, an online survey conducted by the global market research and public opinion specialist (IPSOS), Romanians diagnosed with a chronic illness had an overall greater openness to vaccination (8% declared that they are already immunized, and 57% declared that they are willing to get vaccinated) (IPSOS, 2021). One explanation stems from the fact that the two surveys were conducted in different moments of the pandemic. During the first state of emergency, the present research was done when no vaccine was available and little was known about the SARS-COV-2 virus, while the IPSOS survey was conducted in 2021 after the vaccine started to be available for older adults. Attitudes toward vaccination can change throughout a public health crisis (Fridman et al., 2021). Most likely, subsequent scientific information about vaccination benefits and the pro-vaccination national campaign have encouraged vaccination behavior in people with chronic illness.

Given the shifts mentioned above in attitudes toward vaccination, relatively stable psychological characteristics and behavioral outcomes can represent a valuable baseline for vaccination campaigns and strategies. The primary reasons for deferring vaccination are due to concerns about side effects and safety of the COVID-19 vaccine, lack of trust in the government, and concern that COVID-19 vaccines are developed too quickly (Nguyen et al., 2021), low confidence in the COVID-19 vaccine and the health service response during the pandemic, worse perception of government measures, perception of the information provided as inconsistent and contradictory (Soares et al., 2021).

According to a recent systematic review of vaccine acceptance rates (Sallam, 2021) targeting 33 different countries, more research is needed to identify the mechanisms underlying vaccine hesitancy because low rates of COVID-19 vaccine acceptance were reported worldwide which can eventually represent a general public health issue. The present data shows that the relationship is partially mediated by two components of the HBM model: the perceived threat of disease and the benefits of vaccination. Pandemic worry predicted vaccination intention directly but also through the contribution of the perceived threat of disease and the benefits of vaccination. The result is consistent with previous studies on influenza vaccination and the HBM theoretical framework on predicting COVID-19 health-related behaviors. For example, Liao et al. found in their study that vaccination intention mediated the effect of pandemic worry on vaccination decisions. Also, the perceived threat of disease, benefits, and vaccination barriers mediated the relationship between pandemic worry and vaccination intention during the H1N1 pandemic (Scherr et al., 2016).

This result has potential practical implications for healthcare specialists and policymakers, as it brings to their attention factors that help promote vaccination acceptance and prevent future COVID-19 outbreaks. From a distal perspective, the result pinpoints ways of communicating public messages regarding vaccination. For example, highlighting the disease’s proper impact will prevent people from experiencing unhealthy levels of worry. Presenting evidence of COVID-19 vaccine efficacy and the benefits of having the vaccine (especially for vulnerable groups, such as chronic illness patients) will encourage the population to follow vaccination recommendations and reduce the risk of getting the infection.

One potential issue is whether people must experience a higher level of pandemic worry to impact their vaccination intention. After all, we want people to get vaccinated without experiencing high levels of worry or anxiety. And then, the critical question becomes how we can maintain low levels of pandemic worry and an adequate level of the perceived threat that would still prompt people to vaccinate. Having an adequate level of the perceived threat and highlighting the benefits of COVID-29 vaccination could be enough to engage people in future vaccination behaviors? This topic is worth more exploration.

Knowing that vaccine efficacy and adverse event concerns of the HBM constructs are considered to be significant predictors of COVID-19 vaccination intent (Lin et al., 2020), we conclude that taken together, the findings of the present study provide helpful insight regarding guidance for individually tailored interventions that can use HBM components to raise the level of vaccination intention in a constructive and specific manner.

Notwithstanding the contributions of this research, a limitation of the results derives from the study’s cross-sectional nature, which does not allow for timeline inferences. Longitudinal studies could reveal a better understanding of the investigated phenomena and reinforce potential causal relationships between pandemic worry, the HBM components, and vaccination intention. It is reasonable to assume a temporal order between pandemic worry on the one hand and benefits and barriers to vaccination on the other hand. The assumption is limited without a longitudinal design for pandemic worry and the perceived threat of COVID-19, respectively COVID-19 self-efficacy. The sampling procedure does not allow for the generalization of results. Due to the online data collection, only Internet users could participate, excluding more vulnerable participants with lower academic or financial levels. All the measures used in this study were self-report, and there is the possibility of biased results due to common method variance or social desirability.

## Data Availability Statement

The datasets presented in this study can be found in online repositories. The names of the repository/repositories and accession number(s) can be found below: https://data.mendeley.com/datasets/m7jrfgnwzs/1.

## Ethics Statement

The studies involving human participants were reviewed and approved by Ethics board of the University of Bucharest (notice no. 27/11.06.2021). The patients/participants provided their written informed consent to participate in this study.

## Author Contributions

CI and DI contributed to the conception, the design of the study, and its implementation, and wrote the first draft of the manuscript. EA and DC wrote sections of the manuscript and reviewed the manuscript. All authors read and approved the submitted version.

## Conflict of Interest

The authors declare that the research was conducted in the absence of any commercial or financial relationships that could be construed as a potential conflict of interest.
