# Supplementary material for: COVID-19 Pandemic Worry and Vaccination Intention: The Mediating Role of the Health Belief Model Components
Source: Front Psychol. 2021 Jul 12;12:674018. doi: 10.3389/fpsyg.2021.674018 (PMC8311124; doi:10.3389/fpsyg.2021.674018)
Supplement: Supplementary file 1 [file Table_1.DOCX]

**Supplementary material**

**Table S1** Socio-demographic characteristics of the participants

|  | **Adults without chronic illness (N = 687)** | **Adults with chronic illness (N = 177)** | **χ^2^ (p-value)** |
| --- | --- | --- | --- |
| Gender |  |  | .86 (.35) |
| Female | 452 (65.80%) | 123 (69.50%) |  |
| Male | 235 (34.20%) | 54 (30.50%) |  |
| Education |  |  | 15.7 (.001) |
| Secondary/ vocational school | 41 (5.97%) | 24 (13.55%) |  |
| High-school | 155 (22.56%) | 49 (27.69%) |  |
| Faculty-level education | 262 (38.13%) | 56 (31.64%) |  |
| Master’s degree or higher | 229 (33.34%) | 48 (27.12%) |  |
| Living environment |  |  | .01 (.90) |
| Urban | 560 (81.51%) | 145 (81.92%) |  |
| Rural | 127 (18.49%) | 32 (18.08%) |  |
