# Supplementary material for: COVID-19 Pandemic Worry and Vaccination Intention: The Mediating Role of the Health Belief Model Components
Source: Front Psychol. 2021 Jul 12;12:674018. doi: 10.3389/fpsyg.2021.674018 (PMC8311124; doi:10.3389/fpsyg.2021.674018)
Supplement: Supplementary file 2 [file Table_2.DOCX]

**Supplementary material**

**Table S2** Descriptive statistics on buying behavior and vaccination intention

|  | **Food supplies** | | **Medicine supplies** | | **Sanitary supplies** | | **Vaccination intention** | |
| --- | --- | --- | --- | --- | --- | --- | --- | --- |
|  | **Group 1** | **Group 2** | **Group 1** | **Group 2** | **Group 1** | **Group 2** | **Group 1** | **Group 2** |
| Same amount | 245 (35.66%) | 59 (33.34%) | 406 (59.10%) | 84 (47.46%) | 221 (32.16%) | 52 (29.38%) |  |  |
| Twice as much | 159 (23.14%) | 37 (20.90%) | 127 (18.49%) | 33 (18.64%) | 159 (23.14%) | 29 (16.38%) |  |  |
| Three times as much | 121 (17.62%) | 29 (16.38%) | 69 (10.04%) | 24 (13.56%) | 104 (15.14%) | 25 (14.12%) |  |  |
| Four times as much or more | 162 (23.58%) | 52 (29.38%) | 85 (12.37%) | 36 (20.34%) | 203 (29.55%) | 71 (40.11%) |  |  |
| Yes |  |  |  |  |  |  | 284 (41.34%) | 80  (45.19%) |
| No |  |  |  |  |  |  | 141 (20.52%) | 26 (14.69%) |
| Maybe |  |  |  |  |  |  | 262 (38.13%) | 71 (40.11%) |

Group 1: adults without chronic illness; Group 2: adults with chronic illness.
