# Supplementary material for: COVID-19 Pandemic Worry and Vaccination Intention: The Mediating Role of the Health Belief Model Components
Source: Front Psychol. 2021 Jul 12;12:674018. doi: 10.3389/fpsyg.2021.674018 (PMC8311124; doi:10.3389/fpsyg.2021.674018)
Supplement: Supplementary file 3 [file Table_3.DOCX]

# **Supplementary material 3**

# **Measurement results for the instruments**

# **Pandemic worry (Dispositional Pandemic Worry Scale, adapted after Scherr et al., 2016)**

Principal component analysis revealed that Bartlett’s test of sphericity was significant (χ2 = 5738, df = 28, p < .001), and the KMO index for sample adequacy was .88, which means the main assumptions for PCA were met. As expected, the results of the exploratory factor analysis, with Oblimin rotation, indicate the items load two factors (similar to the original scale), which explain 80.5% of the construct’s cumulative variance. Factor one explains 42.8% of the variance. The first four items load the worry frequency factor, and the remaining four items load the worry severity factor (Scherr et al., 2016). Table S3 presents the factor loadings for each item.

## **Table S3** Factor loadings for pandemic worry scale

|  | | **Factor** | | | |  | |
| --- | --- | --- | --- | --- | --- | --- | --- |
|  | | **1** | | **2** | | **Uniqueness** | |
| 1.I have trouble sleeping because of pictures or thoughts about COVID-19 that come to mind. |  |  |  | 0.846 |  | 0.204 |  |
| 2.I have repetitive thoughts about COVID-19. |  |  |  | 0.747 |  | 0.222 |  |
| 3.I have dreams about COVID-19. |  |  |  | 0.943 |  | 0.258 |  |
| 4.Pictures about COVID-19 pop into my mind. |  |  |  | 0.862 |  | 0.200 |  |
| 5.I am afraid of the physical consequences that COVID-19 infection can have on me. |  | 0.843 |  |  |  | 0.197 |  |
| 6.I worry about my health because of my chances of getting COVID-19. |  | 0.854 |  |  |  | 0.217 |  |
| 7.I feel anxiety when I think of the possible consequences of getting COVID-19. |  | 0.935 |  |  |  | 0.152 |  |
| 8.I keep thinking about the physical consequences of getting COVID-19. |  | 0.964 |  |  |  | 0.108 |  |
| Note: Oblimin rotation was used due to the correlations between the factors. | | | | | | | |
|  | | | | | | | |

# **The perceived threat of COVID-19 (adapted after Champion, 1999)**

Following principal component analysis of the four items, Bartlett’s test of sphericity was significant (χ2 = 1782, df = 6, p < .001), and the KMO index was .75, making the items fit for factorial analysis. Exploratory factor analysis, with Varimax rotation, indicates the items load one factor, which explains 65.2% of the construct’s cumulative variance. Table S4 displays the factor loadings.

## **Table S4** Factor loadings for the perceived threat of COVID-19

|  | | **Factor** | |  | |
| --- | --- | --- | --- | --- | --- |
|  | | **1** | | **Uniqueness** | |
| 1.To what extent you believe you will get a COVID-19 infection in the next 3 months? |  | 0.894 |  | 0.201 |  |
| 2.To what extent you believe your relatives will get a COVID-19 infection in the next 3 months? |  | 0.924 |  | 0.146 |  |
| 3. To what extent you believe your friends will get a COVID-19 infection in the next 3 months? |  | 0.914 |  | 0.164 |  |
| 4. To what extent you believe COVID-19 infection is severe? |  | 0.348 |  | 0.879 |  |
| Note: Varimax rotation was used. | | | | | |
|  | | | | | |

# **The benefits of vaccination (adapted after Champion, 1999)**

The principal component analysis showed that Bartlett’s test of sphericity was significant (χ2 = 2346, df = 10, p < .001), and the KMO index was .84, a good value indicating the usefulness of factor analysis. Exploratory factor analysis indicates the items load one factor, which explains 67.6% of the construct’s cumulative variance. Table S5 presents the factor loadings.

## **Table S5** Factor loadings for benefits of vaccination scale

|  | | **Factor** | |  | |
| --- | --- | --- | --- | --- | --- |
|  | | **1** | | **Uniqueness** | |
| 1.If I get the COVID-19 vaccine, I won’t worry about getting infected. |  | 0.805 |  | 0.352 |  |
| 2.The vaccine can help me prevent a COVID-19 infection. |  | 0.888 |  | 0.211 |  |
| 3.If I get the COVID-19 vaccine, I won’t have severe symptoms if I get infected. |  | 0.841 |  | 0.292 |  |
| 4.Immunization is the best and safest way to prevent COVID-19 infection. |  | 0.686 |  | 0.530 |  |
| 5.The COVID-19 vaccine will significantly decrease the chances of dying because of the virus. |  | 0.873 |  | 0.238 |  |
| Note: Varimax rotation was used. | | | | | |
|  | | | | | |

# **Barriers to vaccination (adapted after Champion, 1999)**

Principal component analysis indicated a significant value for Bartlett’s test of sphericity (χ2 = 3229, df = 45, p < .001), and the KMO index for sample adequacy was .80, which makes the data fit for factor analysis. The results of the exploratory factor analysis, with Oblimin rotation, indicate the items load two factors, which explain 53.9% of the construct’s cumulative variance. Factor one explains 21.4% of the variance, and it reflects various attitudes concerning vaccination. Factor two explains 32.5% of the total variance, and it refers to a lack of knowledge about the COVID-19 vaccine. Table S6 reveals the factor loadings for this scale.

## **Table S6** Factor loadings for barriers to vaccination scale

|  | | **Factor** | | | |  | |
| --- | --- | --- | --- | --- | --- | --- | --- |
|  | | **1** | | **2** | | **Uniqueness** | |
| 1.I’m afraid to get a COVID-19 vaccine due to the possible adverse side effects. |  |  |  | 0.826 |  | 0.199 |  |
| 2.I’m afraid to get a COVID-19 vaccine because I don’t know all the details and its implications. |  |  |  | 0.880 |  | 0.156 |  |
| 3.I don’t know the means and procedures to get a COVID-19 vaccine. |  |  |  | 0.746 |  | 0.526 |  |
| 4.Getting a vaccine is embarrassing. |  | 0.698 |  |  |  | 0.573 |  |
| 5.Getting a COVID-19 vaccine is a waste of time. |  | 0.729 |  |  |  | 0.372 |  |
| 6.Vaccine immunization is painful. |  | 0.622 |  |  |  | 0.613 |  |
| 7.The medical staff doing the vaccine is usually rude. |  | 0.497 |  |  |  | 0.735 |  |
| 8.I will forget the appointment date for the vaccination. |  | 0.754 |  |  |  | 0.460 |  |
| 9.I have other problems more important than getting a vaccine. |  | 0.705 |  |  |  | 0.423 |  |
| 10.I’m too old to expose myself to unnecessary COVID-19 vaccination risks. |  | 0.624 |  |  |  | 0.557 |  |
| Note: Oblimin rotation was used due to the correlations between the factors. | | | | | | | |
|  | | | | | | | |

# **Self-efficacy regarding COVID-19 infection (adapted after Champion et al., 2005)**

After conducting the principal component analysis, Bartlett’s test of sphericity was significant (χ2 = 1096, df = 10, p < .001), and the KMO index was .75, a good value. Exploratory factor analysis suggests the items load one factor, which explains 51.8% of the construct’s cumulative variance. Table S7 presents the factor loadings.

## **Table S7** Factor loadings for self-efficacy scale

|  | | **Factor** | |  | |
| --- | --- | --- | --- | --- | --- |
|  | | **1** | | **Uniqueness** | |
| 1.I am confident that I will be able to protect myself from COVID-19 infection. |  | 0.707 |  | 0.501 |  |
| 2. If necessary, I am confident that I can receive the appropriate medical care. |  | 0.787 |  | 0.381 |  |
| 3. If needed, I am confident that the treatment I will receive for COVID-19 infection will work. |  | 0.698 |  | 0.513 |  |
| 4. I am confident that I can protect my family or loved ones from COVID-19 infection. |  | 0.776 |  | 0.398 |  |
| 5. I am confident that I can procure food and other resources during this period. |  | 0.619 |  | 0.617 |  |
| Note: Varimax rotation was used. | | | | | |
|  | | | | | |
